# Solid oxide fuel cell interconnect design optimization considering the thermal stresses

**DOI:** 10.1007/s11434-016-1146-3

**Published:** 2016-07-20

**Authors:** Min Xu, Tingshuai Li, Ming Yang, Martin Andersson

**Affiliations:** 1 School of Energy Science and Engineering, University of Electronic Science and Technology of China, Chengdu, 611731 China; 2 Department of Energy Sciences, Faculty of Engineering, Lund University, P.O. Box 118, 221 00 Lund, Sweden

**Keywords:** Solid oxide fuel cell, Thermal stresses, Interconnect, Optimization, Finite element method

## Abstract

The mechanical failure of solid oxide fuel cell (SOFC) components may cause cracks with consequences such as gas leakage, structure instability and reduction of cell lifetime. A comprehensive 3D model of the thermal stresses of an anode-supported planar SOFC is presented in this work. The main objective of this paper is to get an interconnect optimized design by evaluating the thermal stresses of an anode-supported SOFC for different designs, which would be a new criterion for interconnect design. The model incorporates the momentum, mass, heat, ion and electron transport, as well as steady-state mechanics. Heat from methane steam reforming and water–gas shift reaction were considered in our model. The results examine the relationship between the interconnect structures and thermal stresses in SOFC at certain mechanical properties. A wider interconnect of the anode side lowers the stress obviously. The simulation results also indicate that thermal stress of coflow design is smaller than that of counterflow, corresponding to the temperature distribution. This study shows that it is possible to design interconnects for an optimum thermal stress performance of the cell.

## Introduction

The solid oxide fuel cell (SOFC) is considered to be one of the most promising new energy technologies for high energy efficiency, environmental friendly and fuel diversity [1, 2]. The performance of an SOFC is strongly related to properties of the electrode materials, seals or interconnect materials and impurities in the fuel [3–7]. During the past decades, there have been significant research aiming to increase the performance of SOFC by exploring new materials or improving synthesis techniques and optimizing the cell structure [8–11]. However, the SOFC technology still faces many challenges, before it could be commercialized, since cost reduction and an extended lifetime are required.

Numerical simulation as an economic approach to design materials, cell or stack structure was used to develop the SOFC technology further. The effect of microstructure on effective ionic and electrical electrode conductivities was investigated by numerical optimization, which determines the effective conductivities and degradation of materials [12]. The mass and heat transport, and electrochemical reaction were considered for SOFC optimization by developing numerical models [13–15]. The mixing ratio of fuel, gas flow rate and reforming reaction, utilizing various fuels were studied to archive an optimized SOFC system. Beside the material structure, heat and mass transfer processes and electrochemical problems, the thermo-mechanical phenomena attracted significant attentions in recent years [16–18]. As the most important part of thermo-mechanical phenomena, thermal stresses occurring to various components in SOFC strongly impact the cell lifetime by inducing cracks, contact loss and structural instability [19].

Thermal stress analysis of a single cell can be used to evaluate the possibility of cracks or flaws that was not able to be detected earlier and easier in experimental works [20]. For a planar anode-supported SOFC, thermal stress and thermal fluid behavior can be analyzed using a 3D integrated numerical model [21]. Maximum principal stress within the cell increases with a high current and temperature gradient, which was in good agreement with the experimental work. Since the flow distribution also had influence on the temperature distribution, thermal stresses of planar SOFC operating at coflow and counterflow were compared [22]. The results indicates that the temperature gradient near the fuel inlet for counter-flow pattern is much larger compared to that of co-flow pattern. Thermal stress in an single cell is different compared to the stress distribution in the stack, i.e., future studies are needed. Jiang et al. [23] built a thermo-electrochemical-structure model combining both the finite-volume and the finite-element approaches to investigate the thermal behavior and the thermal-stress of a SOFC with the bonded compliant seal design. It was found that, the thermal stresses depended on the location and the cell voltage. The second largest stress region within the cell was near the inlet. Except the effects of materials properties, the operating conditions such as the load of cell in a stack should be investigated for practical modeling. Effects of the applied assembly load on the thermal stress distribution in the integrated planar SOFC stack with a compressive sealing design were characterized by a 3D multi-cell model [24]. The results showed that the expansion mismatch rather than the applied compressive load dominated the thermal stress distribution of the cell components. There was also evidence that ceramic components suffered significant stress when subjected to an idealized operating duty cycle [25]. Stresses were generated due to differential thermal expansion of the layers indicating that there is a high probability of failure during these phases of the duty cycle, i.e. when cell is cooled from sintering to room temperature or is heated from room temperature to operating temperature.

However, the just mentioned studies mainly focused on the thermal stresses distribution, considering the multiphysics process or operating conditions but neglecting the interconnect structural effects. As a component for connecting the electrodes and loads, the interconnect is not only essential to provide paths for electron transport in single cell (or to the neighboring cell in stack) and to protect the electrode material from damages in an ambient environment, but can also maintain the thermal mechanical properties matching to the adjacent electro-active components. Therefore, the selection criteria of interconnect materials and optimization of the structure is more important and stringent than other components of the cell. The ceramic interconnect possesses a good stability and retains a fine compatibility with other components. However, the conductivity of ceramics is not appreciable below 600 °C and the poor sinterability of ceramic interconnects is also a challenge for implementation [26]. While, the metallic alloys are low cost, ease of fabricating and high mechanical strength, made it more attractive than ceramic oxides as interconnects in intermediate temperature SOFC stacks [27].

Normally, the interconnect optimization focused on the electrical performance, degradation processes or temperature distribution, since the structure and the shape of interconnects were related to concentration polarization and electric resistance [28–30]. However, the thermal stress depends on the temperature distribution and the load conditions of a complete single cell would be affected by the interconnect structure. With contact area increasing, power density and temperature gradient would be reduced when a decreasing size of collecting pins tended to gain a better temperature homogeneity and power density [31]. Beside the contact area and the size of collecting pins, addition of interconnect ribs is another option for modifying interconnect structure. While increasing the number of interconnect ribs and reducing the gap between them at the cathode side, the lateral conduction distance can be decreased, and an enhancement of the cell performance of more than 30 % can be achieved [32].

Besides, altering the width of ribs is easier both in numerical and experimental design. 3D numerical model was built to analyze the effect of rib width on cell performance [33]. The relationship, between the contact resistance and the optimal rib widths was given as a guidance for an engineering optimization. When considering the size of ribs, the increased width gives a better conduction of the electrical current and reduced ohmic losses, while narrow ribs are needed to facilitate a more uniform distribution of reacting gases and thus promoting the electrochemical performance. This implicates that a tradeoff of rib size to the cell performance is very significant.

In this work, in order to optimize the interconnect structure, based on calculations of the thermal stress field, a 3D comprehensive bi-layer model was developed. Elaborating the reliable strategies for the optimization coupling electrochemical and mechanical properties is of crucial importance for new SOFC designs. Different cell configurations with a geometry design based on the finite element method were applied in this model. The influences from the shape and size of interconnect as well as the components for SOFC involved the thermal stress distribution were investigated in terms of ion/electronic, momentum, mass and heat transport.

## Mathematical model

**Fig. 1:**
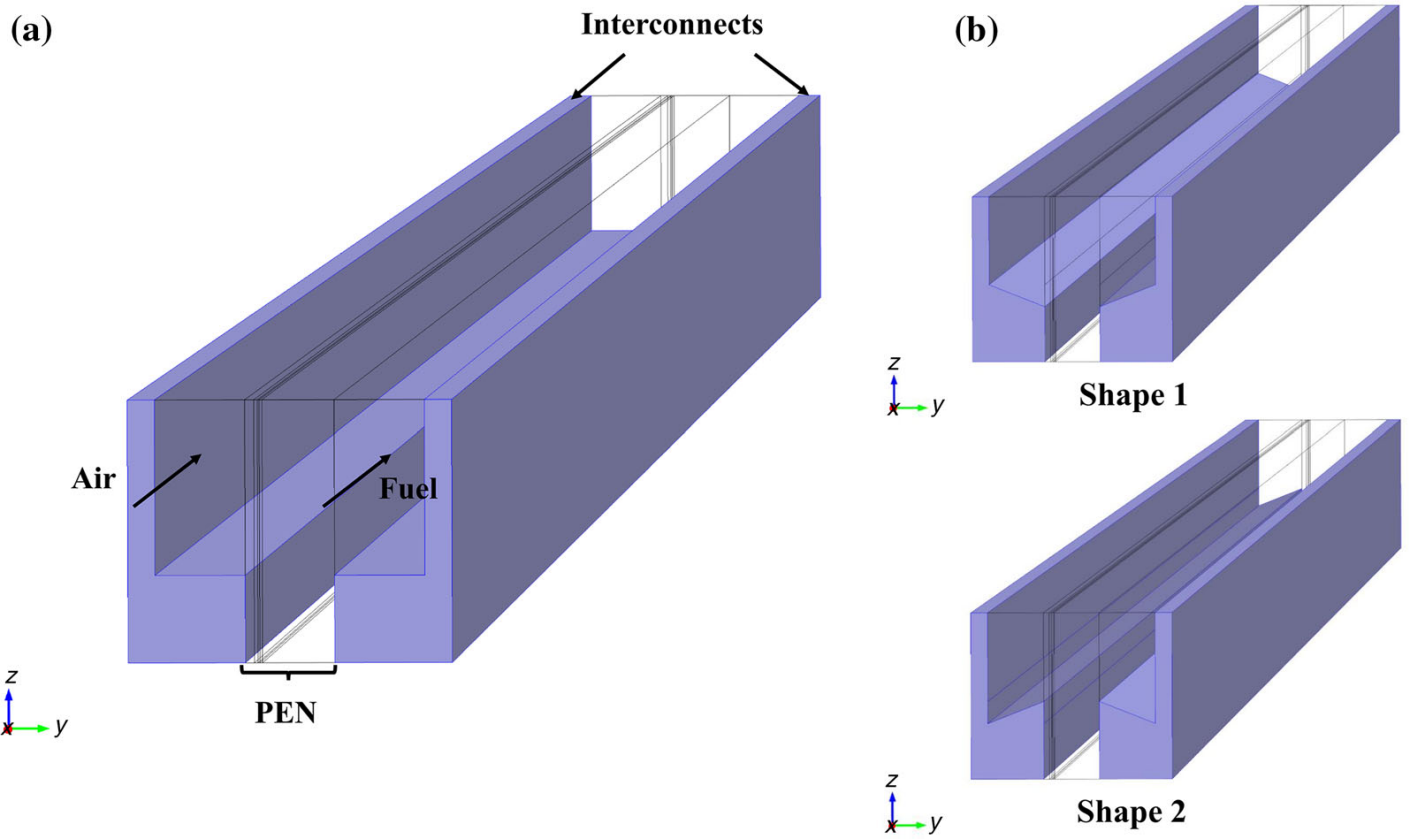
(Color online) Interconnect (lilac color)-design of three cases (not to scale) with **a** standard case (just coflow shown here) and **b** shape 1 and 2, respectively

Conservation and constitution laws were applied to each domain to obtain a comprehensive model containing all the above mentioned phenomena. Stress analysis of an SOFC was performed using the commercial software COMSOL Multiphysics (version 5.0). A half-cell model with bipolar channels operating with humidified hydrogen, carbon monoxide and methane as fuel. The material structure parameters and geometry were selected based on a cell developed and tested at Ningbo Institute of Material Technology & Engineering (NIMTE) [34]. The geometry was built based on the interfacial zone or layer thickness in those experiments [35, 36] and the interfacial layers in the model were assumed to be 15 and 20 μm thickness for active anode and cathode layer and 0.1 m length for the cell, as shown in Fig. 1. Note that, the coflow case was used as a demonstration for our model geometry. The parameters of the geometry are presented in Table 1. Besides, two different interconnects designs are also shown in Fig. 1. The design 1 shows symmetrical interconnects with small contact area with the electrodes, while shape 2 has large contact area with electrodes.

**Table 1:** SOFC cell geometry

Geometry parameter options	Thickness	Direction
Cell length	100 mm	*x*
Gas channel height	0.5 mm	*y*
Gas channel width	1 mm	*z*
Interconnect rib thickness	0.5 mm	*y*
Interconnect thickness	150 μm	*y*
Anode support layer thickness	400 μm	*y*
Anode active layer thickness	15 μm	*y*
Cathode support layer thickness	50 μm	*y*
Cathode active layer thickness	20 μm	*y*
Electrolyte thickness	10 μm	*y*

## Electrochemical model

The reactions considered are shown in Eqs. (1)–(5). Carbon monoxide is oxidized in the electrochemical reaction (Eq. (3)), but reacts faster with water in the water–gas shift reaction (WGSR, Eq. (5)). Methane reacts with steam (MSR) in Eq. (4).

1 $$ \frac{1}{2}{\text{O}}_{2} + 2{\text{e}}^{ - } \to {\text{O}}^{2 - } , $$

2 $$ {\text{H}}_{2} + {\text{O}}^{2 - } \to {\text{H}}_{2} {\text{O}} + 2{\text{e}}^{ - } , $$

3 $$ {\text{CO}} + {\text{O}}^{2 - } \to {\text{CO}}_{2} + 2{\text{e}}^{ - } , $$

4 $$ {\text{CH}}_{4} + {\text{H}}_{2} {\text{O}} \to 3{\text{H}}_{2} + {\text{CO,}} $$

5 $$ {\text{CO}} + {\text{H}}_{2} {\text{O}} \to {\text{H}}_{2} + {\text{CO}}_{2} . $$

## Ion and electron transport

For the ion and electron transport processes, the critical voltage distribution can be described as the potential difference between the anode and cathode current collectors [37]. The operating cell voltage (*E*) is reduced due to the internal resistance and polarizations, as shown in Eq. (6)

6 $$ E = E^{\text{OCV}} - \left( {\eta_{\text{act}} + \eta_{\text{ohm}} + \eta_{\text{conc}} } \right). $$

Here, $E^{\text{OCV}}$ is the open-circuit voltage, *η*_act_, *η*_ohm_, *η*_conc_ the activation, ohmic and concentration overpotentials respectively.

7 $$ \eta_{\text a} = \phi_{\text{s}} - \phi_{\text{l}} - E_{{{\text{eq}},{\text{a}}}} , $$

8 $$ \eta_{\text{c}} = \phi_{\text{s}} - \phi_{\text{l}} - E_{{{\text{eq}},{\text{c}}}} . $$

Here, $\phi$ is the potential and *E*_eq_ the equilibrium voltage. The index “a” and “c” stand for the anode and cathode, respectively. The electrode materials are noted as “s” and the electrolyte material as “l”.

When a hydrogen-steam mixture is used as fuel, the reversible open-circuit voltage, $E^{\text{OCV}}$ in Eq. (6) corresponds to the Gibb’s free enthalpy. $\Delta G_{{{\text{f}},x,T}}$ is the Gibb’s free enthalpy of the oxidation reaction of hydrogen [38]:

9 $$ E^{\text{OCV}} = - \frac{{\Delta G_{{{\text{f}},{\text{H}}_{2} {\text{O}},T}}^{0} }}{2F} + \frac{RT}{2F}\cdot { \ln }\left( {\frac{{p\left( {{\text{H}}_{2} } \right)_{\text{anode}} \sqrt {p\left( {{\text{O}}_{2} } \right)_{\text{cathode}} } }}{{p\left( {{\text{H}}_{2} {\text{O}}} \right)_{\text{anode}} }}} \right), $$

10 $$ \Delta G_{{{\text{f}},{\text{H}}_{2} {\text{O}},T}}^{0} = - 247.4 + 0.0541T. $$

The impact from carbon monoxide and hydrocarbons should be taken into account for a complete model. However, Eq. (10) is sufficient for the study performed in this work with focus on thermal stress.

The activation polarization/current density relationship is described by the Bulter–Volmer equation:

11 $$ i = AVi_{0} \left\{ {\frac{{P_{{{\text{H}}_{2} {\text{O}},{\text{TPB}}}} }}{{P_{{{\text{H}}_{2} {\text{O}},{\text{b}}}} }}{ \exp }\left[ {\frac{{\alpha n_{{{\text{e}},{\text{a}}}} F\eta_{\text{a}} }}{RT}} \right] - \frac{{P_{{{\text{H}}_{2} ,{\text{TPB}}}} }}{{P_{{{\text{H}}_{2} ,{\text{b}}}} }}{ \exp }\left[ {\frac{{\left( {1 - \alpha } \right)n_{{{\text{e}},{\text{a}}}} F\eta_{\text{a}} }}{RT}} \right]} \right\}. $$

Here, *p* is the partial pressure at the triple phase boundary (TPB) or bulk fluid within the gas channels (b) and *n*_e_ is the number of electrons transferred per reaction. In the high current region with sufficiently large *η*_a_ where

12 $$ { \exp }\left[ {\frac{{\alpha n_{{{\text{e}},{\text{a}}}} F\eta_{\text{a}} }}{RT}} \right] \ll { \exp }\left[ {\frac{{\left( {1 - \alpha } \right)n_{{{\text{e}},{\text{a}}}} F\eta_{\text{a}} }}{RT}} \right]. $$

Equation (11) can be rewritten as

13 $$ i = - AVi_{0} \frac{{P_{{{\text{H}}_{2} ,{\text{TPB}}}} }}{{P_{{{\text{H}}_{2} ,{\text{b}}}} }}{ \exp }\left[ {\frac{{\left( {1 - \alpha } \right)n_{{{\text{e}},{\text{a}}}} F\eta_{\text{a}} }}{RT}} \right]. $$

The activation polarization is calculated by using the exchange current density. For the electron and ion transfer problem in the anode and cathode, the charge conservation equation and the governing equations for the ion and electron transport by Ohm’s law are implemented as following:

14 $$ i_{\text{s}} = \nabla \cdot \left( { - \sigma_{\text{s}} \nabla \phi_{\text{s}} } \right), $$

where $\sigma$ is the ion/electron conductivity and $\phi$ is the potential, $i_{\text{s}}$ is the total volumetric current density. In the anode, $i_{\text{s}}$ is the volumetric local current densities of electrochemical reactions of fuel.

The electronic conductivity in the electrodes ($\sigma_{\text{s}}$, within the anode and the cathode) and ionic conductivity in the electrolyte ($\sigma_{\text{l}}$) are calculated as described in Ref. [39]. The actual length that ions and electrons are transported in the electrodes is increased when the effects of the microscopic structures in the material are considered. This is accounted for by using the effective conductivities of each material which were determined by the structure-dependent tortuosities and the volume fractions/porosities [40].

As a medium for ion and transfer from the cathode to anode, the charge conservation in electrolyte can be written as

15 $$ \nabla i_{\text{l}} = Q_{\text{l}} , $$

where $Q_{\text{l}}$ is the source term, in the electrolyte which is equal to zero because there is no production or consumption of ions in electrolyte.

When the heat source $Q_{\text{h}}$ for the ion/charge transport is taken into account, the energy conservation equation from the Joule effect can be expressed as

16 $$ Q_{\text{h}} = \sigma_{\text{l}} \nabla \phi_{\text{l}} \cdot \nabla \phi_{\text{l}} + \sigma_{\text{s}} \nabla \phi_{\text{s}} \cdot \nabla \phi_{\text{s}} . $$

## Momentum, mass and heat transport

The temperature distributions depends on the flow of gases in the channels and porosity of the electrodes that are related to the momentum, mass and heat transfer processes.

The momentum transport in the porous materials as well as in the fuel and air channel are solved simultaneously [41], as

17 $$ \left( {\frac{\mu }{k} + \rho \nabla \cdot \vec{u}} \right) \cdot \vec{u} - \nabla \left[ { - p + \frac{1}{\varepsilon }\left\{ {\psi + \frac{2\mu }{3}\left( {\nabla \cdot \vec{u}} \right)} \right\}} \right] = \varvec{F}. $$

Here, ***F*** is the volume force vector, *p* the pressure, *k* the permeability of the porous material, $\vec{u}$ the velocity vector, $\varepsilon$ the porosity and $\psi$ the viscous stress tensor. The viscosity ($\mu$) and density ($\rho$) for the gas mixtures are dependent on local temperature and the mole fractions and are calculated as described in Ref. [42]:

18 $$ \mu_{i} = \sum\limits_{k = 1}^{7} {b_{k} } \left( {\frac{T}{1{,}000}} \right)^{k} , $$

19 $$ \mu_{g} = \sum\limits_{i} {x_{i} \mu_{i} } , $$

20 $$ \rho_{i} = \frac{{p{\sum }x_{i} M_{i} }}{RT}, $$

where the *b*_*k*_ is the species dependent parameter and ‘‘*k*’’ stands for the number of species dependent parameters in the viscosity equation. *x*_*i*_ mole fraction of species, $M_{i}$ the molecular weight of species *i*.

The mass balance for a gas species, *i*, can be expressed as

21 $$ \rho \left( {u\nabla } \right)\omega_{i} + \nabla J_{i = } R_{i} , $$

in which $J_{i}$ is the mass diffusion flux and $\omega_{i}$ is the mass fraction, $R_{i}$ is the extra source term for production and consumption of species. Note that, $R_{i} = 0$ in the gas channels.

22 $$ R_{i} = \frac{vi}{{n_{i} F}}, $$

$v$ is the stoichiometric coefficient for species, $i$ is the volumetric current density for the electrochemical reaction, and $n_{i}$ is the number of participating electrons in the electrochemical reaction.

In this work, the Maxwell–Stefan model is used because of its simplicity and good accuracy:

23 $$ J_{i} = - \left( {\rho \omega_{i} \mathop \sum \limits_{j} D_{ij} d_{k} + D_{i}^{T} } \right) ,$$

$D_{i}^{\text{T}}$ is the thermal diffusion coefficient which is neglected due to its very small effect here.

A concentration gradient in the porous electrodes is considered when the effects of mass transfer under normal operating conditions for the redox reaction are calculated. In the porous medium, the molecular diffusion and Knudsen diffusion are commonly used to describe the mass diffusion mechanisms. The molecular diffusion is used to describe the situation when the pore size is significantly larger than the mean free path of the gas molecule diffusion [43]. When both Knudsen and molecular diffusion are considered, the effective diffusion coefficients can be calculated as

24 $$ D_{{{\text{eff}},ij}} = \frac{\varepsilon }{\tau }\left( {\frac{{D_{ij} \cdot D_{{{\text{k}},ij}} }}{{D_{ij} + D_{{{\text{k}},ij}} }}} \right) .$$

Here, $\varepsilon$ is the porosity and $\tau$ is the effective tortuosity factor. The effective diffusivity coefficients are calculated assuming electrodes with corrected factor $\frac{\varepsilon }{\tau } = 0.16$. $D_{{{\text{k}},ij}}$ is the Knudsen diffusion coefficient of the component *i* with the component *j* in a gas mixture, which is defined as [44]

25 $$ D_{{{\text{k}},ij}} = \frac{2}{3}r_{\text{e}} \sqrt {\frac{8 \cdot RT}{{\uppi \cdot M_{ij} }},} $$

where $r_{\text{e}}$ is the effective pore radius.

26 $$ M_{ij} = \frac{2}{{\frac{1}{{M_{i} }} + \frac{1}{{M_{j} }}}}, $$

where $M_{i} ,M_{j}$ are the molecular weight of species *i, j,* respectively.

Heat generation within the cell from the electrochemical reactions (electron and ion transport as well as electrode reactions) and the internal steam reforming reaction within the anode are included as source term in the model [45]:

27 $$ r_{\text{MSR}} = AV_{\text{MSR}} \left( {943{ \exp }\left( {\frac{{ - 225 \times 10^{3} }}{R \cdot T}} \right)P_{{{\text{CH}}_{4} }} P_{{{\text{H}}_{2} {\text{O}}}} - 7.74 \times 10^{ - 9} { \exp }\left( {\frac{ - 1937}{R \cdot T}} \right)P_{\text{CO}} P_{{{\text{H}}_{2} }}^{3} } \right), $$

28 $$ r_{\text{WGSR}} = k_{\text{WGSR}} \left( {P_{{{\text{H}}_{2} {\text{O}}}} P_{\text{CO}} - \frac{{P_{{{\text{H}}_{2} }} P_{{{\text{CO}}_{2} }} }}{{k_{\text{WGSR}} }}} \right). $$

The temperature is assumed to be locally equal for the solid material and the gases, based on the minor temperature difference between the gas- and the solid-phase. The energy conservation equation can be written as

29 $$ \rho_{\text{g}} c_{{{\text{p}},{\text{g}}}} \cdot \vec{u} \cdot \nabla T = \nabla \cdot \left( {k_{\text{eff}} \nabla T} \right) + Q_{\text{h}} , $$

Here, *Q*_h_ is the heat generation or consumption, *k*_eff_ is the effective thermal conductivity and *c*_p,g_ is the gas-phase specific heat. The heat source term *Q*_h_, due to electrochemical reactions, ion and electron transport and losses through the activation can be defined as

30 $$ Q_{\text{h}} = i\left( {\frac{{T \cdot\Delta S_{\text{r}} }}{{n_{\text{e}} \cdot F}} + \eta_{\text{act}} } \right) + {\sum }\frac{{i^{2} }}{\sigma } + {\sum }\left( {r_{\text{ref}} \Delta H_{\text{ref}} } \right), $$

where Δ*S*_r_ is the reaction entropy change, *r*_ref_ is the reforming reaction rate and Δ*H*_ref_ is the enthalpy change of the reforming reactions.

## Thermal stress couplings

The materials used in this model are the ones most frequently used for SOFC. Table 2 shows material mechanical properties, which are obtained from literatures [39, 43, 46]. It is noted that the mechanical properties are determined by the content of materials as well as the synthesis process, which is the reason for the difference in properties listed in Table 2. The material properties of the interfacial layers are assumed to be between those of the electrode and the electrolyte respectively. All of the materials are assumed to behave as linear elastic and isotropic. It should be noted that the boundaries of this single cell model are assumed to be free, i.e., load effects are not considered here.

**Table 2:** Material properties used for the calculation of the thermal stress model

Layer	Young’s modulus (GPa)	Poisson’s ratio	CTE (10^−6^ K^−1^)
Anode support layer (Ni–YSZ)	220	0.3	12.5
Cathode support layer (LSM)	114	0.28	12.4
Electrolyte (YSZ)	205	0.3	10.3
Active anode layer	213	0.3	11.4
Active cathode layer	160	0.3	11.4
Interconnect (stainless steel)	205	0.28	12.3

CTE thermal expansion coefficient, YSZ yttria-stabilized zirconia, LSM strontium doped lanthanum manganite

SOFC components expand with temperature, causing thermal strains to develop in the material when the deformation is constrained. The overall strain results from the summation of elastic and thermal stresses, i.e., the initial contributions are neglected:

31 $$ \varepsilon = \varepsilon_{\text{el}} + \varepsilon_{\text{th}} $$

The form of strain as follow

32 $$ \varepsilon_{\text{el}} = \left( {\varepsilon_{xx} , \varepsilon_{yy} , \varepsilon_{zz} , \gamma_{yz} ,\gamma_{xz} , \gamma_{xy} } \right), $$

here *ε*_*xx*_, *ε*_*yy*_, *ε*_*zz*_, *γ*_*yz*_, *γ*_*xz*_, *γ*_*xy*_ are the longitudinal and shear components for strain, respectively. The thermal strain depends on the temperature, *T*, the stress-free reference temperature, *T*_ref_, and the coefficient of thermal expansion (CTE), *α*:

33 $$ \varepsilon_{\text{th}} = \alpha \left( {T - T_{\text{ref}} } \right). $$

Determining the stress free temperature is critical as it directly affects the magnitude of the thermal stress induced in the material. For SOFCs it is widely accepted that *T*_ref_ is the sintering temperature, at which different layers are joined.

The stress–strain relationship for the linear material was calculated as

34 $$ \sigma = D\varepsilon_{\text{el}} + \sigma_{0} , $$

where *σ*_0_ is the initial stress, which is treated as the residual stress in model. The elasticity matrix (*D*) for isotopic material is defined as,

35 $$ D = \frac{E}{{\left( {1 + v} \right)\left( {1 - 2v} \right)}}\left[ {\begin{array}{*{20}c} {\begin{array}{*{20}c} {1 - v} & v & v \\ \end{array}      \begin{array}{*{20}c} 0 & 0 & 0 \\ \end{array} } \\ {\begin{array}{*{20}c} v & {1 - v} & v \\ \end{array}      \begin{array}{*{20}c} 0 & 0 & 0 \\ \end{array} } \\ {\begin{array}{*{20}c} v & v & {1 - v} \\ \end{array}      \begin{array}{*{20}c} 0 & 0 & 0 \\ \end{array} } \\ {\begin{array}{*{20}c} 0 & 0 & 0 \\ \end{array}      \begin{array}{*{20}c} {\frac{1 - 2v}{2}} & 0 & 0 \\ \end{array} } \\ {\begin{array}{*{20}c} 0 & 0 & 0 \\ \end{array}      \begin{array}{*{20}c} 0 & {\frac{1 - 2v}{2}} & 0 \\ \end{array} } \\ {\begin{array}{*{20}c} 0 & 0 & 0 \\ \end{array}      \begin{array}{*{20}c} 0 & 0 & {\frac{1 - 2v}{2}} \\ \end{array} } \\ \end{array} } \right], $$

where *E* is the Young’s modulus and *v* the Poisson’s ratio of the material.

## Boundary conditions and solution methods

The gas inlet velocities are defined as laminar flow profiles, and the boundary condition at the impermeable walls is a nonslip condition for the velocity. At the outlets, the pressure (1.013 × 10^5^ Pa) is fixed. The fuel inlet fractions are defined as 30 % pre-reformed natural gas (as defined by International Energy Agency (IEA), the mole fractions of the fuel are: H_2_: H_2_O: CH_4_:CO: CO_2_ = 0.263 : 0.493 : 0.171 : 0.0294 : 0.0436) were used as fuel in this model. Note that the ion and electron transport was considered as a testing part in our model, i.e., the H_2_ mixture and not the 30 % pre-reformed natural mixture was considered in this section, which is sufficient for the study on thermal stress. The air inlet is defined as air, including oxygen and nitrogen. The boundary conditions for the outlets are defined as convective fluxes. The inlet gas temperature is defined by the operating temperature (1,000 K) and the outlet is defined as a convective flux. The boundaries at the top and the bottom walls of the cell are defined as the symmetry conditions, because it is assumed that the cell is surrounded by other ones with identical temperature distribution. Also the boundaries at the side walls are defined as symmetry conditions, because the channel section is assumed to be surrounded by identical channel sections. The potential at the anode current collector is set to zero and the one at the cathode current collector as the cell operating voltage (0.7 V). All other boundaries and interfaces are electrically insulated.

The governing equations are segregated in five different groups:

1. Velocity field, pressure distribution, and pressure corrections.
2. Temperature distribution.
3. Ion and electron distribution.
4. Mass fraction distribution on the air side and fuel side.
5. Thermal stress distribution.

The segregated solver is applied for 7,136,654 degrees of freedom and the solution tolerance is defined to 0.001 for each segregated group. The calculation time is around 80 h on a single computer with 32 GB RAM and a CPU with 4 GHz. Note that it is hard to give an exact value for the calculation time since the model is built in several steps, where each step starts its calculation from the previous one.

## Results and discussion

Note that the color legend and the operating parameters are kept the same for all cases. The bi-layer SOFC model geometry for a half-cell (symmetry exists in the single cell) includes interconnects with the other components of a SOFC, as illustrated in Fig. 1a. The gas flow direction of coflow case is pointed out with an arrow, while the fuel flow is in the opposite direction for the counterflow case. The experimental data from Kyushu University are used for some adjustment of the simulation conditions as well as validation for the model results, as shown in our previous work [45].

For optimization of the shape, two types were designed to investigate the support and contact effects of interconnects, as shown in Fig. 1b. The rib in shape 1 has an inverted trapezoidal, with the same width of ribs contacting to the PEN structure (support positive layer-active positive layer-electrolyte- support negative layer-active negative layer) as the standard case, but width of ribs linked to the top interconnects increased by 0.1 μm. With the shape 1, the trapezoidal ribs that contact to the PEN structure is wider than the standard case (0.1 μm), as shown in shape 2. Those two types were designed, considering the possibility that the contact area and the mechanical strength of the structure would affect the thermal stress distribution.

**Fig. 2:**
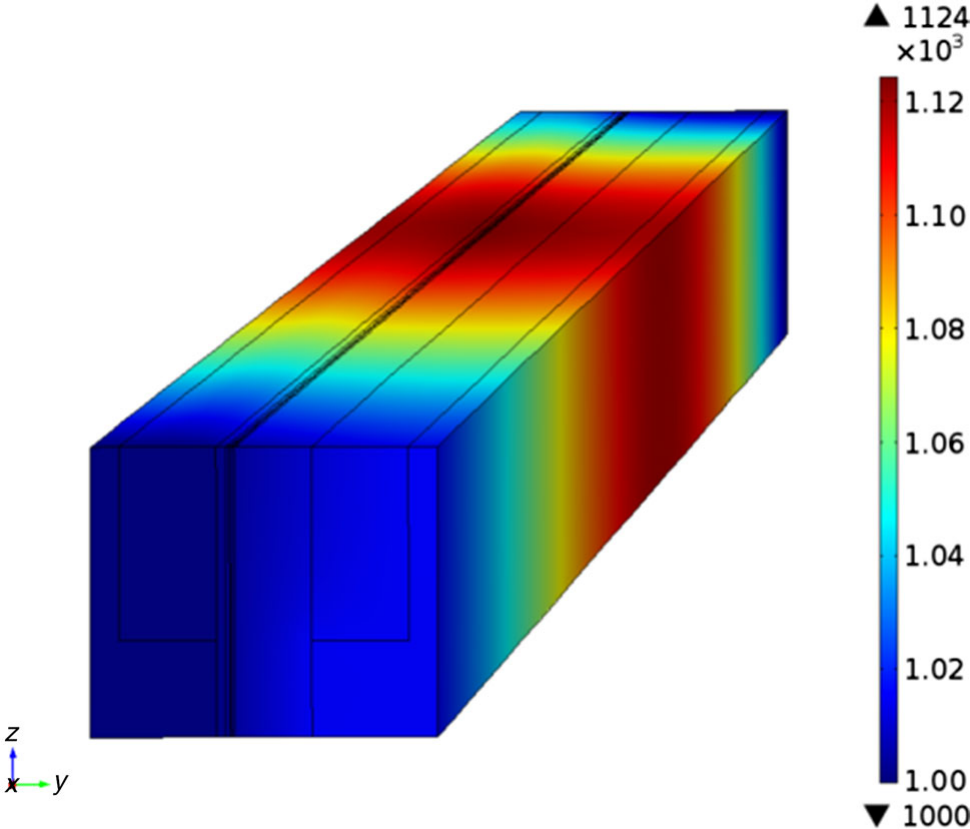
(Color online) Temperature (K) distribution for the counterflow case of standard shape

The temperature distribution for the counterflow case of standard shape is presented in Fig. 2. The temperature increase along the main flow direction is caused by the strongly exothermic electrochemical reactions as well as from the different polarizations. Note that the temperature decrease since the endothermic reforming reactions are considered. The maximum temperature (1,124 K) is located in the inside of the cell near to the position the fuel inlet (0.04 m to the fuel inlet).

**Fig. 3:**
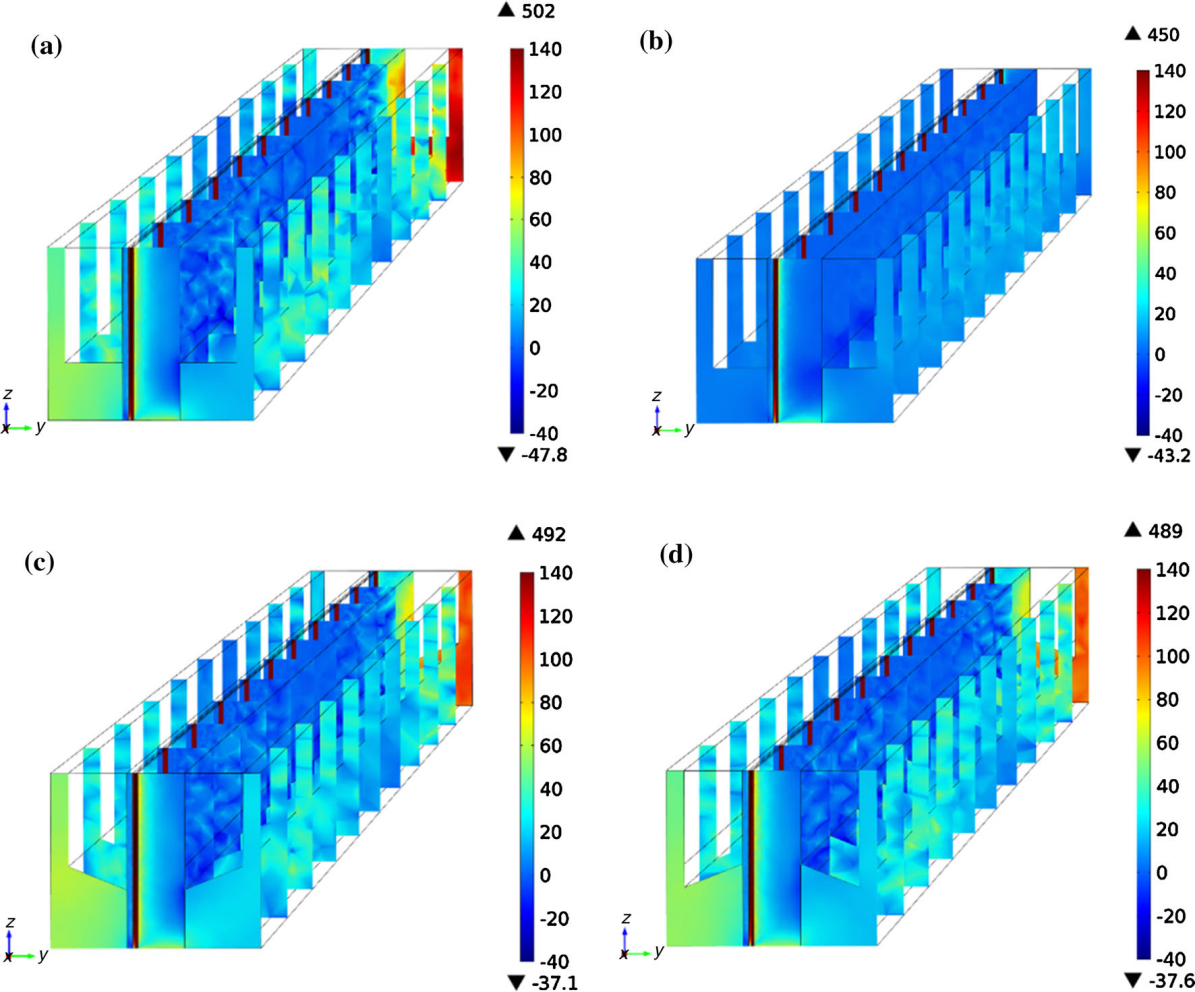
(Color online) First principal stresses (MPa) of **a** counterflow of standard shape, **b** coflow of standard shape, **c** counterflow shape 1 and **d** counterflow shape 2

Coupling the mass and heat transport, reforming reaction, solid mechanical and the first principal stress of SOFC with different designs are shown in Fig. 3. The positive values tell that tensile stress and negative value for compressive stress. The active electrodes and electrolyte component involved in a tensile stress while the support electrode for a compressive stress. This stress difference results from the properties mismatch and it may lead to curling of the components. It is noted that maximum tensile and compressive stresses for coflow are smaller than that for counterflow, corresponding to the temperature distribution. The greater temperature gradient in counterflow also results in an obviously tensile stress for interconnect at fuel inlet, as seen in Fig. 3a. Similar stress distribution can be seen in shape 1 and 2, shown in Fig. 3c and d. The stress decreases with the new interconnect shape design, and the shape 2 is slightly smaller than shape 1, which may be due to the subtle difference in temperature distribution of heat conduction for the two interconnect structures.

**Fig. 4:**
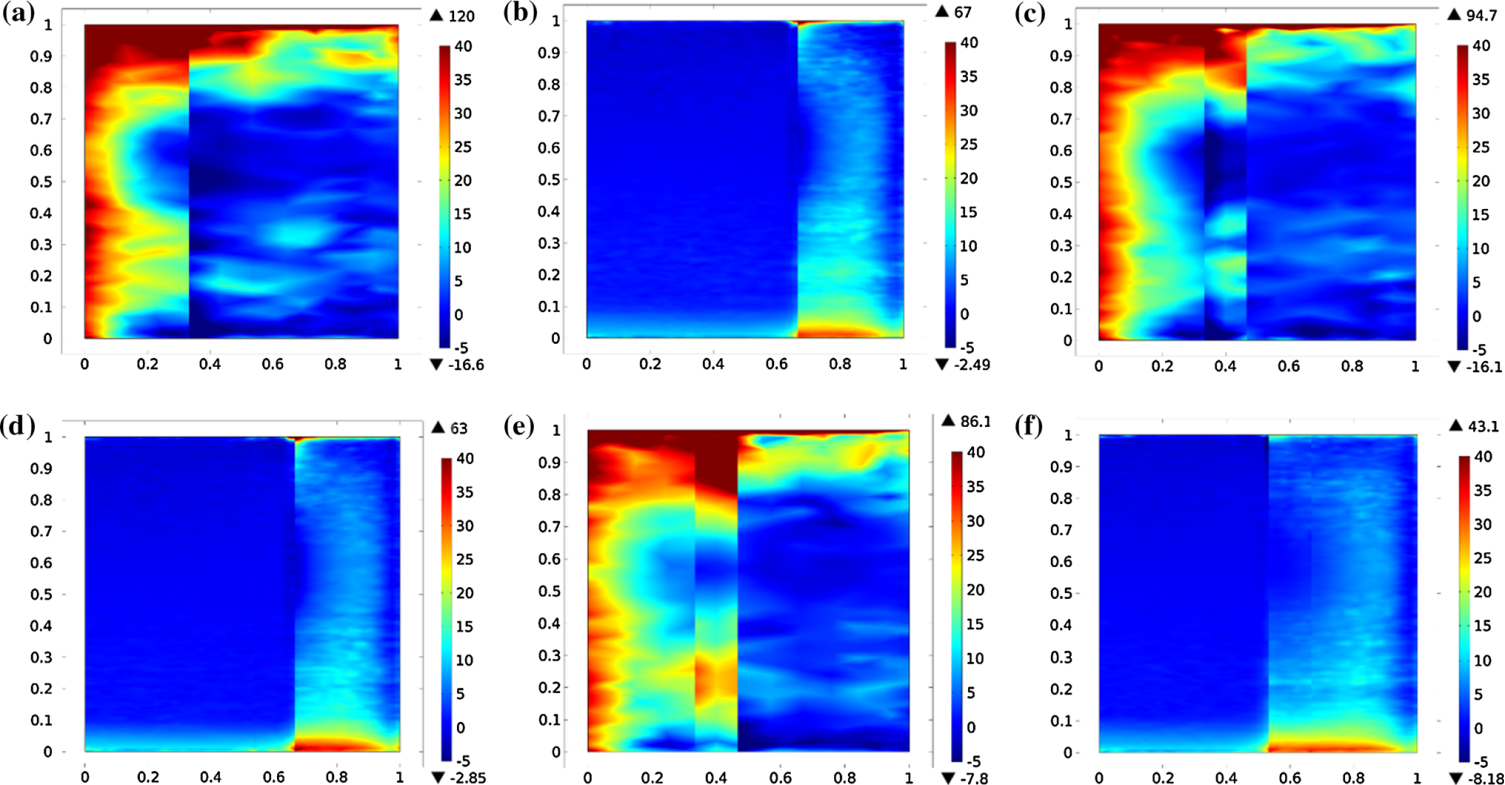
(Color online) First principal stresses (MPa) of counterflow **a** standard case for interface of interconnect and anode, **b** standard case for interface of interconnect and cathode, **c** shape 1 for interface of interconnect and anode, **d** shape 1 for interface of interconnect and cathode, **e** shape 2 for interface of interconnect and anode and **f** shape 2 for interface of interconnect and cathode

The effects of mismatch mechanical properties can be analyzed by comparing the stress distribution at each components’ interface. Since the mismatch between the interconnect and the cathode is larger than for the anode (Table 2), the thermal stress at cathode side is larger than at anode side, as shown in Fig. 4. It should be noted that, the horizontal direction represents the *z* axis in the model (direction vertical to gas flow) and the vertical direction represents the *x* axis (direction parallel to gas flow). The palliation effects of thermal stress of shape 1 and 2 design was obvious. The interconnect shape design is more effective to reduce the tensile stress in the PEN structure. However, the thermal stress distribution would be extended in the shape 2 design case, because the interconnect has a higher heat conduction compared to the gas, leading to a higher temperature on the surfaces of support layers.

**Fig. 5:**
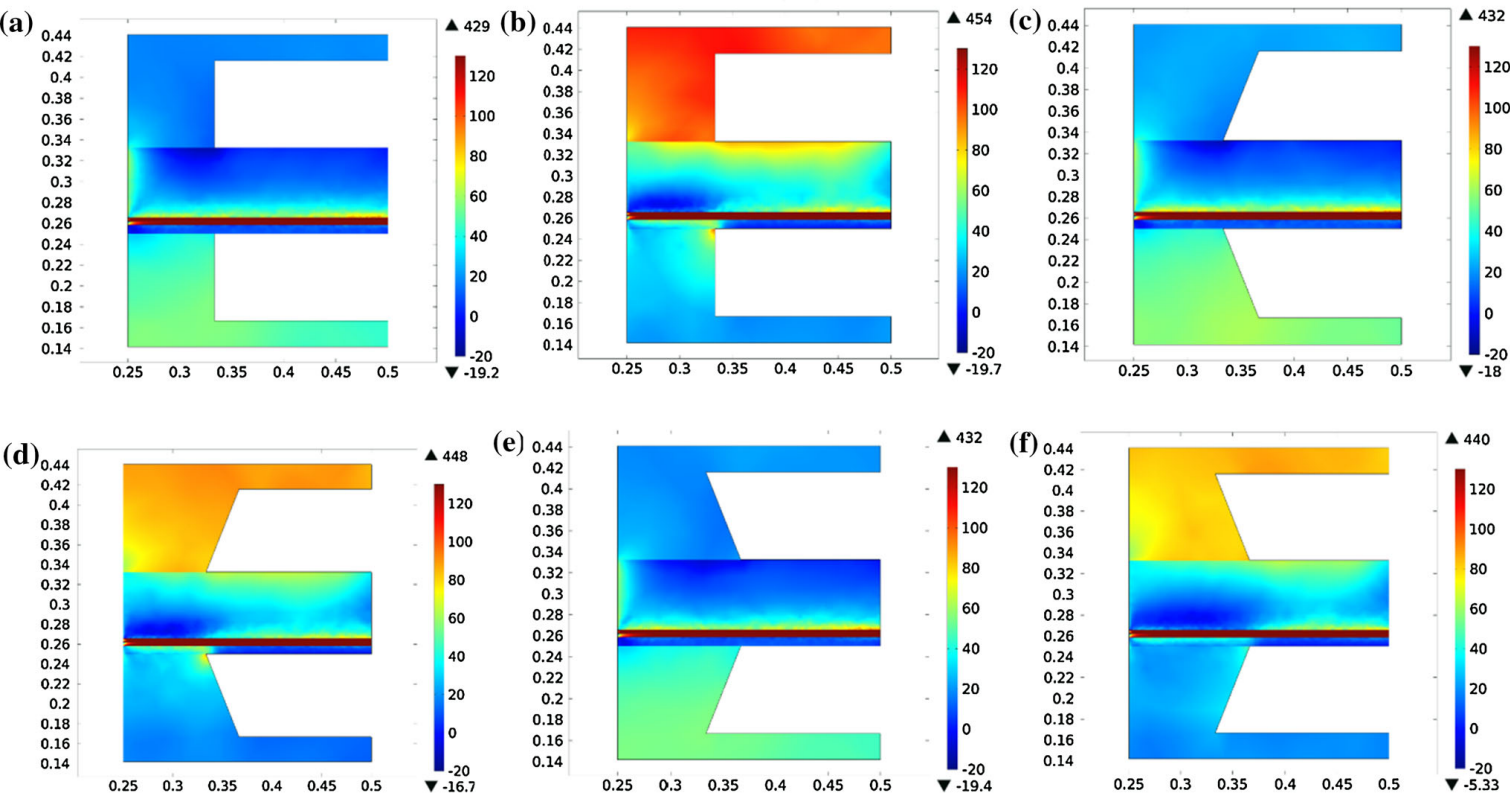
(Color online) First principal stresses (MPa) of counterflow **a** standard case air inlet, **b** standard case fuel inlet, **c** shape 1 air inlet, **d** shape 1 fuel inlet, **e** shape 2 air inlet and **f** shape 2 fuel inlet

The thermal stress on the interface close to the inlet and outlet for the different designs is shown in Fig. 5. Since the temperature gradient at air inlet is smaller, a tiny change of stress can be seen in Fig. 5a, c, e. The slight increase of stress may be due to the slightly increased temperature. However, the tensile stress of the interconnect at the anode side was obviously reduced, and this decrease can also be seen at the active layers and in the electrolyte, compared to the fuel inlet in Fig. 5b, d, f. An increased compressive stress can also be observed within the support layers.

**Fig. 6:**
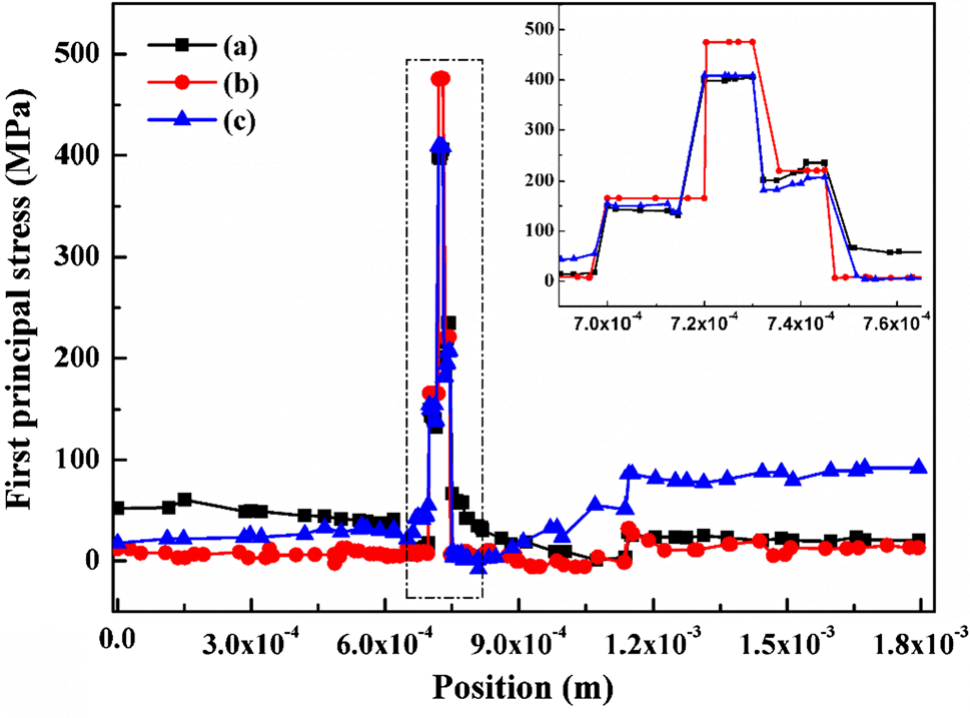
(Color online) First principal stress (MPa) of counterflow case along with the direction vertical to gas flow of air inlet (a), middle part of cell (b) and fuel inlet (c)

Figure 6 shows that the stress has a relationship with the location, i.e. for different components. For the interconnect at the cathode side, the stress at the air inlet is bigger than that at the fuel inlet, and the minimum stress locates at the middle position of the cell. However, the maximum stress belongs to the fuel inlet for the interconnect at the anode side, and stress at air inlet is close but slightly higher than that at the middle position of cell. When considering the thermal stress distribution for the PEN structure, the middle position dominates at the electrolyte while the fuel inlet and air inlet are comparatively large at the cathode and anode support layers, as depicted in the embedded figure.

**Fig. 7:**
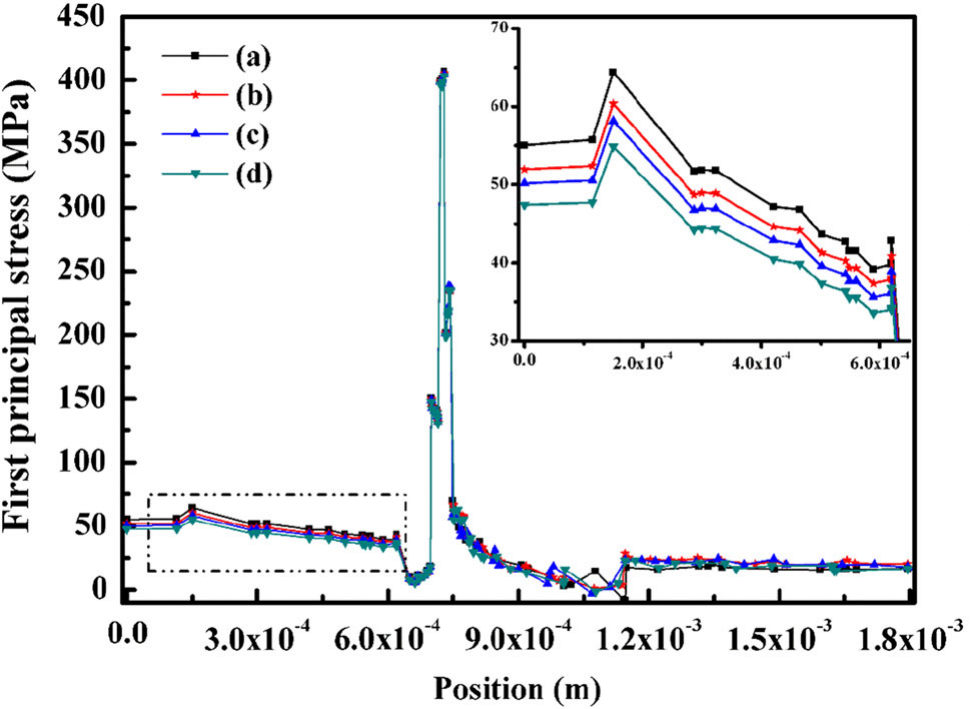
(Color online) First principal stress (MPa) of counterflow case along with the direction vertical to gas flow of air inlet. Standard case (a), interconnect of anode side heighten with 10 μm (b), 15 μm (c) and 20 μm (d)

It is observed in Fig. 7 that the thermal stress is almost the same when the height of rib at anode side is increased, but causes a rise at the interconnect close to the cathode side, which is illustrated in the figure embed in Fig. 7. This may be explained by the symmetry stress effect of structure.

**Fig. 8:**
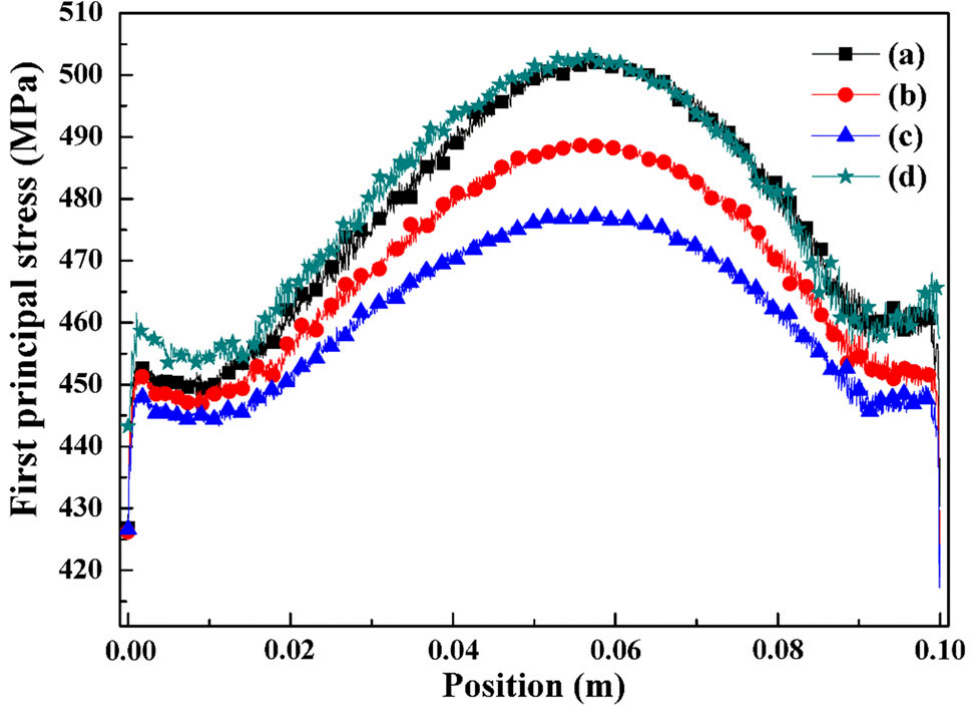
(Color online) Counterflow case first principal stress (MPa) for electrolyte standard case (a), interconnect rib of anode side wider 0.1 mm (b), 0.2 mm (c), and interconnect rib of cathode side wider 0.1 mm (d)

Figure 8 shows that the strong influence of the rib width on the thermal stress. Wider ribs and ribs covering bigger fraction of the cell may reduce the interface resistance to current flow by increasing the electrode–interconnect contact area and reducing the current path through the possibly high resistance electrode material. However, the gas channel would be narrowed, as a consequence, the electrochemistry reaction is affected. To illustrate, considering the three designs in Fig. 8, wider interconnect at the anode side would lower the stress. Conversely, this may be useless for reducing the stress at the cathode side.

**Fig. 9:**
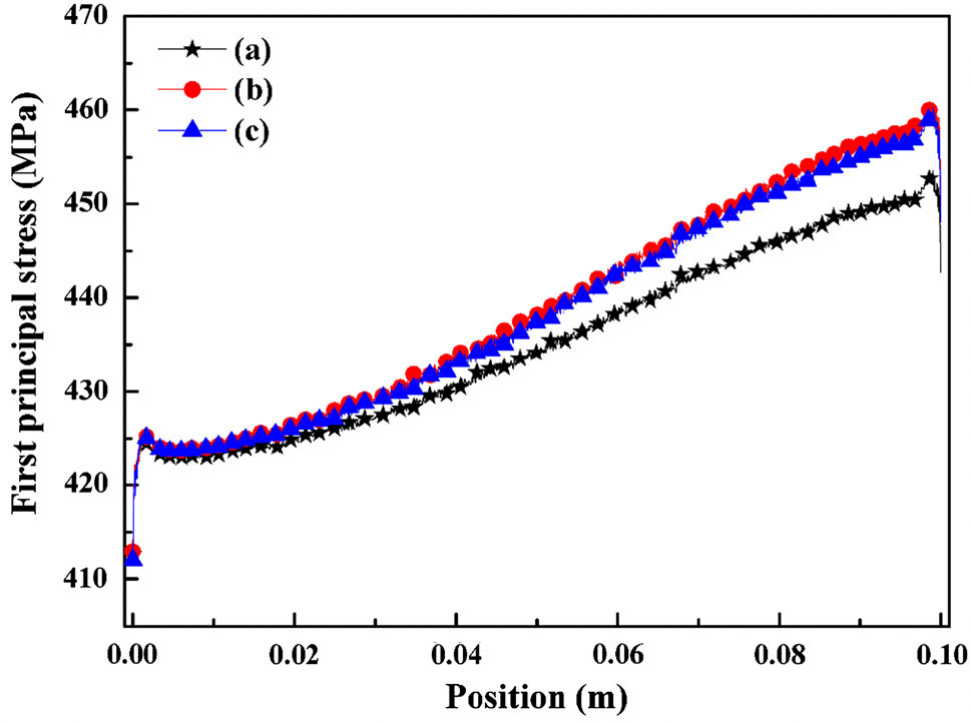
(Color online) First principal stresses (MPa) of coflow case for electrolyte standard case (a), interconnect rib anode wider 0.1 mm (b) and interconnect rib cathode wider 0.1 mm (c)

The thermal stresses of the coflow case for an electrolyte with a wider rib shown in Fig. 9. One can see that the stress obviously rise with wider interconnect ribs. The stress changes increases along the flow because of the rise of temperature. This difference may occur due to the superposition of interconnect and electrodes side (ribs) changes, which affect gas concentration at the three-phase boundary (TPB) region.

## Conclusions

Interconnects are critical to minimize the overall stack resistance and weight as well as to enhance the stability when thermal stresses are considered. A comprehensive model to investigate the relationship between the cell structure, temperature distribution and thermal stresses in SOFCs at certain mechanical properties was built using the finite element method. Proper design of interconnect in conjunction with single-cells were implemented based on the thermal stress optimization performed in this work.

The palliation of thermal stress of different designs was obvious. The interconnect shape design was the most efficient to reduce the tensile stress in PEN structure. Besides, thicker interconnect ribs causes a rise at the interconnect close to cathode side while this effect for thermal stress at the anode side is smaller. It is also observed that, wider interconnect of anode side lowers the stress. We show, by numerical calculations, that the thermal stress can be reduced through structural correlations. This optimization can be used as a new strategy for interconnect optimization in SOFCs which is beyond single criterion for electrochemical performance.

## Appendix: List of symbols

*AV*: Active area to volume ratio (m^2^/m^3^)
*c*_p_: Specific heat at constant pressure (J/(kg K))
*D*: Diffusion coefficient (m^2^/s)
*D*_*i*_^T^: Thermal diffusion coefficient (kg/(m s))
*E*: Actual voltage (V)
*E*: Young modulus (Pa)
*E*_a_: Activation energy (J/mol)
*E*_0_: Ideal voltage before partial pressure consideration (V)
*E*_eq_: Equilibrium voltage (V)
*E*^OCV^: Ideal voltage after partial pressure consideration (V)
***F***: Volume force vector (N/m^3^)
*F*: Faraday constant (96,485 C/mol)
Δ*H*: Enthalpy change of reaction (J/mol)
*i*: Current density (A/m^2^)
*i*_0_: Exchange current density (A/m^2^)
$J_{i}$: Mass diffusion flux
*k*: Thermal conductivity (W/(m K))
*M*_*j*_: Molecular weight of species *j* (kg/mol, g/mol)
*n*_e_: Number of electrons transferred per reaction
*p*: Pressure (Pa or bar)
*Q*_h_: Source term (heat) (W/m^3^)
*r*: Reaction rate (mol/(m^3^ s))
*r*_e_: Pore radius (m)
*R*: Gas constant (8.314 J/(mol K))
$R_{i}$: Extra source term for production and consumption of species
*S*_*i*_: Source term (mass) (kg/(m^3^ s))
Δ*S*: Entropy change of reaction (J/(K mol))
*T*: Temperature (K)
*T*_ref_: Stress-free reference temperature (K)
$$ \vec{u} $$: Velocity vector (m/s)
***u***: Displacement vector
*V*: Volume fraction
*w*_*i*_: Mass fraction of species *i* (kg/kg)
*x*, *y*: Coordinate system (m)
*x*_*j*_: Mole fraction of species *j* (mol/mol)

## Greek symbols

*α*: Coefficient of thermal expansion (K^−1^)
*β*: Transfer coefficient
*ε*: Porosity
*ε*_el_: Elastic stresses contribution
*ε*_th_: Thermal strain contribution
*η*: Over potential (or polarization) (V)
*Φ*: Potential (V)
*k*: Permeability (m^2^)
*μ*: Dynamic viscosity (Pa s)
*ρ*: Density (kg/m^3^)
*σ*_l,s_: Ionic/electronic conductivity (Ω^−1^ m^−1^)
*σ*: Stress
*v*: Poisson’s ratio
*τ*: Tortuosity
Δ: Numerical difference

## References

[1] Atkinson A, Barnett S, Gorte RJ (2004). Advanced anodes for high-temperature fuel cells. Nat Mater.

[2] Park S, Vohs JM, Gorte RJ (2000). Direct oxidation of hydrocarbons in a solid-oxide fuel cell. Nature.

[3] Aljaberi AD, Irvine JTS (2013). Ca-substituted, A-site deficient perovskite La_0.2_Sr_0.7_TiO_3_ as a potential anode material for SOFCs. J Mater Chem A.

[4] Reddy AA, Goel A, Tulyaganov DU (2014). Thermal and mechanical stability of lanthanide-containing glass–ceramic sealants for solid oxide fuel cells. J Mater Chem A.

[5] Jensen SH, Sun X, Ebbesen SD (2016). Pressurized operation of a planar solid oxide cell stack. Fuel Cells.

[6] Li TS, Xu M, Gao C (2014). Investigation into the effects of sulfur on syngas reforming inside a solid oxide fuel cell. J Power Sources.

[7] Xu M, Li B, Wang B (2015). Mechanism of phosphorus and chlorine passivating a nickel catalyst: a density functional theory study. Electrochim Acta.

[8] Chen Y, Zhou W, Ding D (2015). Advances in cathode materials for solid oxide fuel cells: complex oxides without Alkaline earth metal elements. Adv Energy Mater.

[9] Chen Y, Lin Y, Zhang Y (2014). Low temperature solid oxide fuel cells with hierarchically porous cathode nano-network. Nano Energy.

[10] Andersson M, Yuan J, Sundén B (2014). SOFC cell design optimization using the finite element method based CFD approach. Fuel Cells.

[11] Chen D, Zhang Q, Lu L (2016). Multi scale and physics models for intermediate and low temperatures H^+^-solid oxide fuel cells with H^+^/e^−^/O^2−^ mixed conducting properties: part A, generalized percolation theory for LSCF-SDC-BZCY 3-component cathodes. J Power Sources.

[12] Pecho O, Stenzel O, Iwanschitz B (2015). 3D microstructure effects in Ni–YSZ anodes: prediction of effective transport properties and optimization of redox stability. Materials.

[13] Janardhanan VM, Deutschmann O (2011). Modeling diffusion limitation in solid-oxide fuel cells. Electrochim Acta.

[14] Ni M (2013). Modeling and parametric simulations of solid oxide fuel cells with methane carbon dioxide reforming. Energy Convers Manag.

[15] Xie Y, Xue X (2012). Multi-scale electrochemical reaction anode model for solid oxide fuel cells. J Power Sources.

[16] Greco F, Frandsen HL, Nakajo A (2014). Modelling the impact of creep on the probability of failure of a solid oxide fuel cell stack. J Eur Ceram Soc.

[17] Laurencin J, Delette G, Lefebvre-Joud F (2008). A numerical tool to estimate SOFC mechanical degradation: case of the planar cell configuration. J Eur Ceram Soc.

[18] Peksen M (2015). Numerical thermomechanical modelling of solid oxide fuel cells. Prog Energy Combust Sci.

[19] Boccaccini DN, Sevecek O, Frandsen HL (2016). Investigation of the bonding strength and bonding mechanisms of SOFCs interconnector–electrode interfaces. Mater Lett.

[20] Fleischhauer F, Terner M, Bermejo R (2015). Fracture toughness and strength distribution at room temperature of zirconia tapes used for electrolyte supported solid oxide fuel cells. J Power Sources.

[21] Chiang LK, Liu HC, Shiu YH (2010). Thermal stress and thermo-electrochemical analysis of a planar anode-supported solid oxide fuel cell: effects of anode porosity. J Power Sources.

[22] Fan P, Li G, Zeng Y (2014). Numerical study on thermal stresses of a planar solid oxide fuel cell. Int J Therm Sci.

[23] Jiang TL, Chen MH (2009). Thermal-stress analyses of an operating planar solid oxide fuel cell with the bonded compliant seal design. Int J Hydrog Energy.

[24] Lin CK, Huang LH, Chiang LK (2009). Thermal stress analysis of planar solid oxide fuel cell stacks: effects of sealing design. J Power Sources.

[25] Clague R, Marquis AJ, Brandon NP (2012). Finite element and analytical stress analysis of a solid oxide fuel cell. J Power Sources.

[26] Mahato N, Banerjee A, Gupta A (2015). Progress in material selection for solid oxide fuel cell technology: a review. Prog Mater Sci.

[27] Wu J, Liu X (2010). Recent development of SOFC metallic interconnect. J Mater Sci Technol.

[28] Lin Z, Stevenson JW, Khaleel MA (2003). The effect of interconnect rib size on the fuel cell concentration polarization in planar SOFCs. J Power Sources.

[29] Huang CM, Shy SS, Lee CH (2008). On flow uniformity in various interconnects and its influence to cell performance of planar SOFC. J Power Sources.

[30] Stygar M, Brylewski T, Rękas M (2012). Effects of changes in MOLB-type SOFC cell geometry on temperature distribution and heat transfer rate in interconnects. Int J Heat Mass Transf.

[31] Grondin D, Deseure J, Zahid M (2008). Optimization of SOFC interconnect design using multiphysic computation. Comput Aided Chem Eng.

[32] Noh H-S, Hwang J, Yoon K (2013). Optimization of current collection to reduce the lateral conduction loss of thin-film-processed cathodes. J Power Sources.

[33] Kong W, Gao X, Liu S (2014). Optimization of the interconnect ribs for a cathode-supported solid oxide fuel cell. Energies.

[34] Li TS, Gao C, Xu M (2014). Effects of PH_3_ and CH_3_Cl contaminants on the performance of solid oxide fuel cells. Fuel Cells.

[35] Holtappels P, Bagger C (2002). Fabrication and performance of advanced multi layer SOFC cathodes. J Eur Ceram Soc.

[36] Li TS, Xu C, Chen T (2010). Chlorine contaminants poisoning of solid oxide fuel cells. J Solid State Electrochem.

[37] Zhu H, Kee RJ (2008). Modeling distributed charge-transfer processes in SOFC membrane electrode assemblies. J Electrochem Soc.

[38] Holtappels P, Stimming U, Vielstich W, Gasteiger HA, Lamm A (2010). Solid oxide fuel cells (SOFC). Handbook of fuel cells.

[39] Andersson M, Yuan J, Sundén B (2013). SOFC modeling considering hydrogen and carbon monoxide as electrochemical reactants. J Power Sources.

[40] Sohn S, Nam JH, Jeon DH (2010). A micro/macroscale model for intermediate temperature solid oxide fuel cells with prescribed fully-developed axial velocity profiles in gas channels. Int J Hydrog Energy.

[41] Le Bars M, Grae Worster M (2006). Interfacial conditions between a pure fluid and a porous medium: implications for binary alloy solidification. J Fluid Mech.

[42] Andersson M, Yuan J, Sundn B (2012). SOFC modeling considering electrochemical reactions at the active three phase boundaries. Int J Heat Mass Transf.

[43] COMSOL Multiphysics Version 5.0 User Guide. Stockholm, Sweden, 2014

[44] Yuan J, Huang Y, Sundén B (2009). CFD approach to analyse transport phenomena coupled chemical reactions relevant for methane reformers. Heat Mass Transf.

[45] Andersson M, Nakajima H, Kitahara T (2014). Comparison of humidified hydrogen and partly pre-reformed natural gas as fuel for solid oxide fuel cells applying computational fluid dynamics. Int J Heat Mass Transf.

[46] Liu L, Kim G-Y, Chandra A (2010). Modeling of thermal stresses and lifetime prediction of planar solid oxide fuel cell under thermal cycling conditions. J Power Sources.

[47] Xu M, Andersson M, Li TS (2015). Modeling of solid oxide fuel cell with anisotropic conductivity. ECS Transact.

